# *Stellera chamaejasme L.* extracts in the treatment of glioblastoma cell lines: Biological verification based on a network pharmacology approach

**DOI:** 10.3389/fonc.2022.962970

**Published:** 2022-08-17

**Authors:** Kaiyue Wang, Zengyong Wang, Zhiqiang Wang, Xiaoli Xie, Lanlan Zang, Lijuan Wang, Fengyuan Che

**Affiliations:** ^1^ Guangzhou University of Chinese Medicine, Guangzhou, China; ^2^ Department of Neurology, Linyi People’s Hospital, Shandong University, Linyi, China; ^3^ Central Laboratory, Linyi People’s Hospital, Shandong University, Linyi, China; ^4^ Key Laboratory of Neurophysiology, Linyi, China; ^5^ Key Laboratory of Tumor Biology, Linyi, China; ^6^ Clinical Pharmacological Laboratory, Linyi People’s Hospital, Shandong University, Linyi, China; ^7^ Department of Hematology, Linyi People’s Hospital, Shandong University, Linyi, China

**Keywords:** *Stellera chamaejasme* L., glioblastoma, network pharmacology, molecular docking, MAPK9, oxidative stress, Wnt/β-catenin, p62/Nrf2

## Abstract

**Background:**

*Stellera chamaejasme* L (RXLD) has been demonstrated with good clinical effects and medicinal value in the treatment of cancer *in vivo* and *in vitro*. Specifically, RXLD can eliminate aggregation accumulation, which is depicted as a vital characteristic feature of intracranial tumors. The potential pharmacological mechanisms of anti-glioblastoma (GBM) have not been adequately identified.

**Methods:**

The 3D structures of the chemical ingredients in RXLD were imported into the PharmMapper database to construct the pharmacophore models. The gene targets of GBM were obtained from databases. The pharmacophore-targets network and the protein-protein interactions (PPI) were constructed using the String database and were visualized by using Cytoscape. Gene ontology (GO) and Kyoto Encyclopedia of Genes and Genomes pathway (KEGG) enrichment analyses were conducted using Bioconductor software. Cytoscape visualized the relationship of pathways and candidate genes to screen for key target genes. Software packages PyMOL, AutoDock, and Vina acquired the molecular docking results. *In vitro* experiments were undertaken to characterize RXLD extracts’ effects on A172 cell line proliferation, viability, apoptosis, cell cycle, cell wound healing, cell migration, reactive oxygen species generation, and mitochondrial membrane potential. The expression of core genes in the related pathways was detected by Western blotting.

**Results:**

We identified 216 potential targets associated with GBM. The core components in RXLD were neochamaejasmin A, wikstrol A, isochamaejasmin, chamaejasmine, and subtoxin A. The undertaken GO enrichment analysis revealed that oxidative stress, cell proliferation, cell cycle, cell invasion, and cell migration were involved in the biological processes. The KEGG enrichment analysis revealed that the crucial pathway was MAPK pathway, while HRAS, PRKCB, MAPK9, CCND1, and TP53 were distributed in core locations. A total of seven RXLD pharmacophores demonstrated strong spontaneous docking activities with MAPK9. *In vitro* assays indicated that RXLD can induce apoptosis, block the cell cycle in the G2/M and S phases, inhibit cell migration *via* the Wnt/β-catenin pathway, and inhibited p62/Nrf2 pathway.

**Conclusions:**

We speculate that the RAS/MAPK pathway might be an upstream pathway through which the RXLD exerts its anti-GBM effects and might be able to regulate further the Wnt/β-catenin, the oxidative stress, and the ferroptosis pathways.

## Introduction

Gliomas are tumors originating from glial or glial precursor cells and represent the most commonly encountered primary malignant intracranial tumors (1). According to the Central Brain Tumor Registry of the United States, gliomas account for about 25.2% and 82.7% of all central nervous system (CNS) and CNS malignant tumors, respectively. Unfortunately, the glioma with the highest incidence is glioblastoma (GBM; WHO IV), accounting for 58.4% of the CNS malignant tumors (2). In China, the GBM annual incidence is 5−8/100,000, increasing with age. The median age of a new GBM diagnosis is 64 years (3), and the 1-year and 5-year survival rates are 30% and 13%, respectively (4). Due to their high heterogeneity and invasiveness, malignant cells in GBM are difficult to remove completely (5). In contrast, residual tumor cells present with stem cell characteristics with a potential self-updated ability (6), further leading to the establishment of resistance against chemotherapy and radiotherapy (7), thereby resulting in the recurrence of such tumors and the establishment of a refractory disease (8). As a result, GBM treatment is rarely beneficial when using existing treatment methods (9).

Chinese medicine follows the principle of treating based on pattern differentiation and treating both the root and branch (10); thereby, clinical practice has proved that traditional Chinese medicine is a prominent system of therapeutic methods with a long history. According to “Huangdi Neijing (Yellow Emperor’s Inner Classic),” intracranial tumors exert the morphological characteristics and pathological processes of aggregation accumulation (AA), leading to congested stagnation of brain orifices, abnormal transport of spiritual machines, and obstructed brain and body fluid circulation (11), and finally resulting in symptoms such as headache, dizziness, epilepsy, hemiplegia, and hydrocephalus (12). Ai Juan et al. (13) have discovered that Chinese medicine *Angelica sinensis* (Danggui) and *Poria* (Fuling) had been used to treat AA during the Qing Dynasty; however, these herbs are currently only exploited in supplementary anti-cancer strategies. For example, the *Angelica sinensis* polysaccharide has been shown to induce apoptosis and inhibit the invasion of the U251glioma cells through the phosphoinositide 3-kinase (PI3K) / protein kinase B (AKT) pathway. However, it has limited clinical applicability due to its high effective concentrations (400−800 mg/L) (14). Therefore, the monitoring of new traditional Chinese herbs for the treatment of AA is imperative for developing novel glioma treatments.

*Stellera chamaejasme* L. (RXLD), also known as heartbroken grass, steamed bread flower, and Reijiaba (Tibetan medical name), is a Chinese ethnic medicine named RXLD, which refers to the root of RXLD (15). *Stellera chamaejasme* L. is an accepted name as per “The Plant List” (http://www.theplantlist.org) accessed on 2022/5/23. RXLD was first recorded in “Shen Nong’s Materia Medica.” Its taste is bitter and pungent, and its nature is flat. It enters the lung, spleen, and liver meridians (16). As recorded in "Tibetan Materia Medica Tibetan Medicine Volume" (17), Tibetan medicine is mostly used for treating visceral tumors, plagues, and external treatment of bruises.

As recorded in “Compendium of Materia Medica,” RXLD has a strong toxicity and can eliminate phlegm and AA (18). RXLD is itself toxic, but its preparation into a decoction (with vinegar, milk, or wine) can reduce its toxicity and enhance its pharmacological efficacy (19). Ma *et al. (* 20) have found that both the tumor inhibition ratio of an S180 transplanted tumor and the expression levels of caspase-3 were significantly greater than those of the RXLD crude drug groups after using the RXLD wine decoction treatment. RXLD ethanol extracts inhibit hepatocellular carcinoma cell proliferation, induce cell apoptosis, and limit cell invasion through blocking the JAK1/STAT3 pathway by upregulating the expression of miR-134-5p (21) *in vitro*. RXLD exert an eligible therapeutic effect in rat bladder cancer by inducing Fas pathway-related cell apoptosis and inhibiting VEGF regulated tumor angiogenesis *in vivo* (22).Therefore, we hypothesized that RXLD has an eligible medicinal value, and its anti-tumor effect is worth exploring.

The 154 compounds isolated from RXLD were classified as flavonoids, coumarins, lignans, diterpenoids, volatile oils, and other compounds (23). Because of the multitude of ingredients, the anti-tumor mechanism of RXLD involves multiple targets and pathways (24). In this study, we first employed network pharmacology and molecular docking methods to screen for potential pharmacological ingredients in RXLD and for key targets and signaling pathways in the treatment of GBM. Secondly, we prepared RXLD extracts to verify these molecular mechanisms *in vitro*. We found that RXLD extracts can inhibit GBM cell proliferation and induce apoptosis. It can also affect the development of glioma through pathways regulating the cell cycle, the response to oxidative stress, and ferroptosis.

## Materials and methods

### Network pharmacology method

#### Construction of a pharmacophore model containing active RXLD ingredients

Following a literature screening, we obtained the chemical structural information of the 2D and 3D structures as well as the SDF formats of 154 RXLD ingredients by using the PubChem database, the ChemDRAW software, and the Chem3D software. We then put the above-mentioned structural formulas into the PharmMapper database to obtain their pharmacophore models. We subsequently used the UNIPROT database (https://www.uniprot.org/) to annotate the active targets of each of the obtained pharmacophore models.

#### Screening of GBM targets

We searched for “Glioblastoma” in the human gene database GeneCards (https://www.genecards.org/), in the human Mendelian genetics database OMIM (Online Mendelian Inheritance in Man; https://www.omim.org/), in the DNA sequence database GenBank (https://www.ncbi.nlm.nih.gov/genbank/), in the pharmaceutical genomics knowledge base PharmGKB (https://www.pharmgkb.org/), and in the treatment target database TTD (http://db.idrblab.net/ttd/) to obtain the GBM-related disease targets.

#### Drug active ingredients-disease network

The filter condition of “Normfit ≧ 0.8” was applied to explore the core pharmacophore models in the ingredients of RXLD. We then used the screening results to associate them with the GBM targets, thereby constructing and visualizing the relationship web map between the disease targets and the pharmacologically active ingredients by analyzing the key pharmacological active ingredients and their related targets for GBM treatment through Cytoscape 3.8.0.

#### Construction and analysis of protein-protein interaction (PPI) network

The RXLD-related GBM targets were imported into the String database (https://string-db.org/) in order to construct the PPI network. Subsequently, the proteins characterized by “medium confidence ≧ 0.9” were selected for further analysis. The PPI network was topologically analyzed by the CytoNCA plugin of the Cytoscape software, from which the proteins with betweenness (BC), closeness (CC), degree (DC), eigenvector (EC), LAC and network (NC) values greater than the median values, were selected. The nodes in the PPI networks represented the target proteins, while the edges represented the relationships with the targets. The bigger the size and the darker the color of the node in the network, the more significant the target was. After two topological analyses, we obtained the PPI network that highlighted the key RXLD-related GBM targets.

#### Gene ontology (GO) and Kyoto Encyclopedia of Genes and Genomes pathway (KEGG) enrichment analysis

We used the R programming language (R x64, version 4.0.3) and the Bioconductor packages (ColorSpace, Stringi, GgPlot2, Dose, ClusterProfiler, EnrichPlot) to process the GO enrichment analysis. The top 10 results obtained by the GO analysis with filter conditions “P-Value < 0.05, Q-Value < 0.05” were further visualized by a bar chart consisting of the cellular component (CC), the biological process (BP), and the molecular function (MF). We used Strawberry Perl to process the KEGG enrichment signaling pathway. We set the filter conditions “P-Value < 0.05, Q-Value < 0.05” to obtain the key signaling pathways. The key targets/signaling pathways were built and visualized using the Cytoscape software.

#### Molecular docking

We set the potential pharmacological ingredients in the PPI network and the KEGG network as small molecule ligands, and the key RXLD-related GBM targets as the receptors. We then downloaded the 3D structure of these receptors from the Protein Data Bank (https://www.rcsb.org/), then removed water and small molecular ligands (by using the PyMOL software) to prepare the receptors. Themol2 files were built by using the Chem3D 19.0 software. Finally, we wrote the PDBQT files of the receptors and ligands, determined the receptors’ active pockets using the AutoDock 4.2.6 software, and processed the molecular docking using the Vina software. After the docking, we selected the lowest binding energy as the best docking model, and we used the PyMOL software to visualize the docking results.

## Materials

### Cell lines used

The U251, TG905, and A172 cell lines were purchased from the Chinese Academy of Sciences Cell Board and Keygen Biotech.

### Samples preparation

RXLD was purchased from the Bozhou Medicine Market (Anhui, China)and was identified by Dr. YuYun Fan from the Department of Traditional Chinese Medicine in Linyi People’s Hospital. All RXLD samples were powdered and passed through a 100-mesh sieve before extraction. The RXLD powders (20 g) were extracted with 100 ml of 75% ethanol overnight at room temperature. The extracts were centrifuged at 3,500 rpm for 10 min, and the supernatants were collected. Then we applied a set of device named rotary evaporation, which is consists of a low temperature coolant circulation pump (DLSB-5L/10, Gongyi, China), a heating bath (BUCHI, B-100, Switzerland), a rotary evaporator (BUCHI, R-100, Switzerland), and a vacuum pump (BUCHI, I-100, Switzerland) , to separate the ethanol from RXLD extracts. Undergoing the rotary evaporation at a low vacuum condition, the ethanol was evaporated efficiently and the ingredients in the drug were not damaged. The extracts were dried using a vacuum drier (YIHENG17, Shanghai, China) overnight, and frozen by a lyophilizer (BIOCOOL, Beijing, China) afterwards. Finally, we obtained the extracts as powder, and the yield was 30%. The final extracts were dissolved in dimethyl sulfoxide (DMSO) in order to formulate a 100 mg/ml stock solution for use in subsequent experiments.

### Cell counting kit-8 (CCK-8) cell proliferation assays

Cell proliferation assays were completed by using a CCK-8 assay (BestBio, Shanghai, China). Human GBM cell lines A172, TG905, and U251 were seeded into 96-well plates at a density of 3,500 cells/well and incubated overnight before treatment with different concentrations of the RXLD samples. After 24−96 h, the CCK-8 reagent was added to each well, and the absorbance values were measured by using an M5 microplate reader (Molecular Devices, USA) at the wavelength of 450 nm. Finally, we used GraphPad Prism 8.0 software to construct the drug-efficient relationship of the tested drugs and calculate the IC_50_ value.

### Cellular apoptosis assays

Apoptosis assays were conducted by using the annexin V-fluorescein isothiocyanate (FITC)/propidium iodide (PI) double staining apoptosis detection kit (BestBio, Shanghai, China) according to the manufacturer’s instructions. Firstly, the cells were seeded into 6-well plates at a density of 2× 10^5^ cells/well and incubated overnight before treatment with different concentrations of the RXLD samples. After incubating the RXLD samples for 48 h, the cells and their supernatant were harvested and washed twice with ice-cold phosphate-buffered saline (PBS). After centrifugation allowed us to discard the supernatant, the cells were resuspended in PBS and incubated with FITC at 4°C for 15 min, and then incubated with PI at 4°C for 5 min, centrifuged once again in order to discard the supernatant, and then finally resuspended in PBS prior to the analysis *via* flow cytometry (within 1 h).

### Cell cycle assays

Cells were seeded into 6-well plates at a density of 1× 10^6^ cells/well and were incubated overnight before treatment with different concentrations of the RXLD samples. After being treated with RXLD samples for 48 h, the cells were harvested and subjected to the following assays. According to the manufacturer’s instructions, cell cycle assays were conducted using a cell cycle detection kit (BestBio, Shanghai, China). The cells were washed twice with ice-cold PBS and were fixed in 70% ethanol at 4°C overnight before being centrifuged to discard the supernatant. After having their precipitation washed twice with ice-cold PBS, the cells were resuspended in PBS and incubated with RNase A at 37°C for 30 min, and then incubated with PI at 4°C for 1 h (while avoiding exposure to light). The cell cycle distribution was assessed by flow cytometry (BD).

### Cell wound healing assays

Cells were seeded into 6-well plates at a density of 5× 10^5^ cells/well. When the cell confluence reached 80-90% overnight, we used a 200-µL pipette tip to draw a line in the middle of each well. We washed the cells with PBS and incubated them with different concentrations of the RXLD samples and with serum-free medium for 48 h. The cell healing results were observed by microscopy at 0, 6, 12, 24, and 48 h after the addition of the RXLD samples, while ImageJ and GraphPad were used for analysis.

### Cell migration assays

Cells were seeded into 6-well plates at a density of 2 × 10^5^ cells/well and were incubated overnight before their treatment with different concentrations of the RXLD samples for 48 h. The cells were suspended by serum-free medium and then transferred to the upper layer of the transwell chamber (while a complete medium was added to the lower chamber) and cultured 24 h. Finally, the cells were fixed using a multi-formaldehyde solution, stained with crystal purple, and then observed and pictured under the microscope.

### Reactive oxygen species (ROS) assays

Cells were seeded into 12-well plates at a density of 8 × 10^4^ cells/well and were incubated overnight before their treatment with different concentrations of the RXLD samples. After incubating the RXLD samples for 48 h, we harvested the cells and stained them with a 2’,7’-dichlorofluorescein (DCF) probe according to the ROS kit (BestBio, Shanghai, China) instruction manual. We finally employed flow cytometry for the assessment of the active oxygen species assay.

### Mitochondrial membrane potential (JC-1) assays

Cells were seeded into 6-well plates at a density of 2 × 10^5^ cells/well and were incubated overnight before their treatment with different concentrations of the RXLD samples. After incubation of the RXLD samples for 48 h, we harvested the cells and employed the JC-1 probe to stain the cells according to the JC-1 mitochondrial membrane potential detection kit (BestBio, Shanghai, China) instructions. The stained cells were then observed under a fluorescence microscope.

### Western blotting analysis

The cells were seeded into 6-well plates at a density of 5 × 10^5^ cells/well and were incubated overnight prior to their treatment with the RXLD samples. After 48 h, the cells were harvested and lysed on ice, and their proteins were extracted according to the instructions of the protein extraction kit (BestBio, Shanghai, China). Protein concentrations were determined using the bicinchoninic acid (BCA) protein assay kit. After determining the protein concentrations, the extracted lysates were denatured by a sodium dodecyl sulfate (SDS) loading buffer. Equal amounts of protein from the lysates were separated by the SDS-PAGE and transferred onto polyvinylidene fluoride (PVDF) membranes. Membranes were blocked with 5% non-fat milk in TBST (TBS+Tween) and were incubated with primary antibodies at 4°C, overnight. Subsequently, the membranes were washed and incubated with secondary antibodies for 1 h, then washed and visualized by using chemiluminescence (ECL, Amersham-Pharmacia, Uppsala, Sweden). Finally, glyceraldehyde 3-phosphate dehydrogenase (GAPDH) was used as an internal loading control.

### Statistical analysis

All experiments were performed in triplicates and repeated at least twice for each experiment. Two-group comparisons were analyzed for variation and significance using the Student’s *t*-test or the Pearson χ^2^ test. All data shown are mean ± standard deviation (s.d.), while Pearson’s correlation coefficient was also used to measure the correlation of the gene co-expression.

## Results

### Construction of pharmacophore models of active RXLD ingredients

By putting 154 chemical ingredients of RXLD into the PharmMapper database, we obtained 97 kinds of pharmacophore models, of which 39, 17, 14, 20, and 7 corresponded to flavonoids, coumarins, diterpenoids, lignans, and volatile oils and other ingredients, respectively (**Supplementary 1**). In total, 734 targets were obtained by the chemical structures in the pharmacophore models, which might represent the drug targets.

### RXLD-related GBM targets network analysis

**Figure 1:**
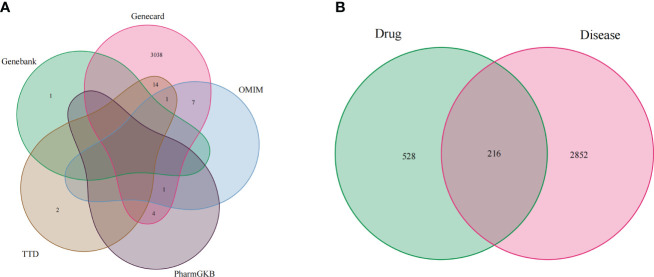
Venn diagram: **(A)** Venn diagram for GBM targets from databases; **(B)** RXLD-related GBM targets’ Venn diagram.

**Figure 2:**
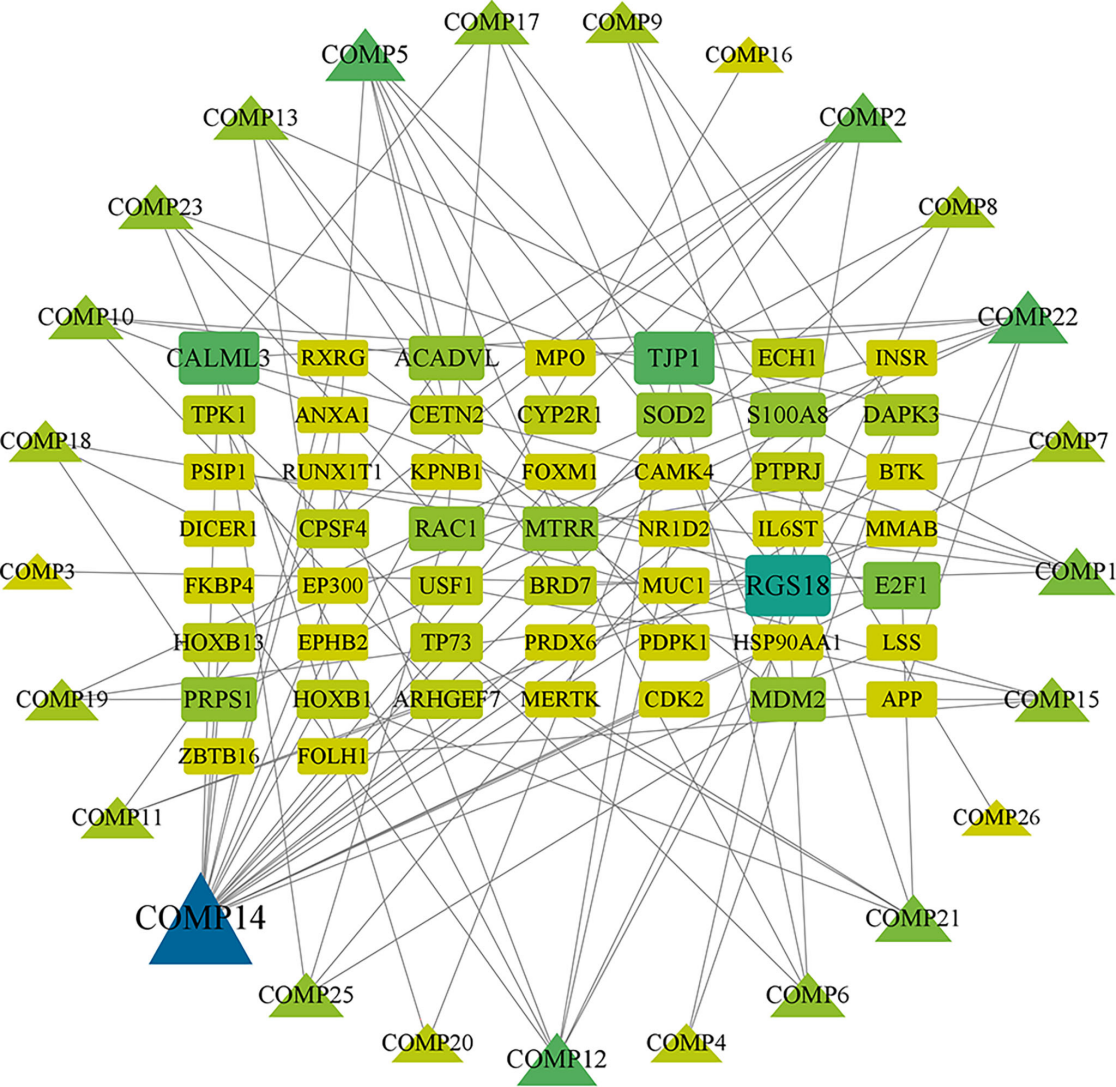
RXLD-related GBM targets’ network. The symbols that are larger and linked to more edges represent targets of higher importance in the network.

Firstly, we merged the GBM-related disease targets obtained from the various databases (3,068 targets) (**Supplementary 2**) and constructed a Venn diagram for GBM targets (**Figure 1A**). Secondly, we combined the pharmacophore models of the RXLD active ingredients with the GBM targets to build the RXLD-related GBM targets’ Venn diagram, a process that returned 216 intersection targets (**Figure 1B**). The RXLD-related GBM targets’ network (**Figure 2**) (**Supplementary 3**, **4**) consisted of 78 nodes and 111 edges, wherein the nodes represented 26 compounds (**Table 1**) and 52 genes. Among them, the degrees of sphondin (COMP14), neochamaejasmin A (COMP5), wikstrol A (COMP12),subtoxin A (COMP22), isochamaejasmenin (COMP2), and chamaejasmine (COMP1) were greater than or equal to 5; as a result, these compounds were identified as the most important compounds in this network. RGS18, Calml3, E2F1, MDM2, and RAC1 were the top 5 targets in gene nodes. The above results showed the above-mentioned active ingredients and targets were in the hub position in the network.

**Table 1:** Active ingredients identified in RXLD and their chemical structures (NormFit≧0.8).

Species	compound ID	Name	Structure	compound ID	Name	Structure
Flavonoids	COMP1	Chamaejasmine	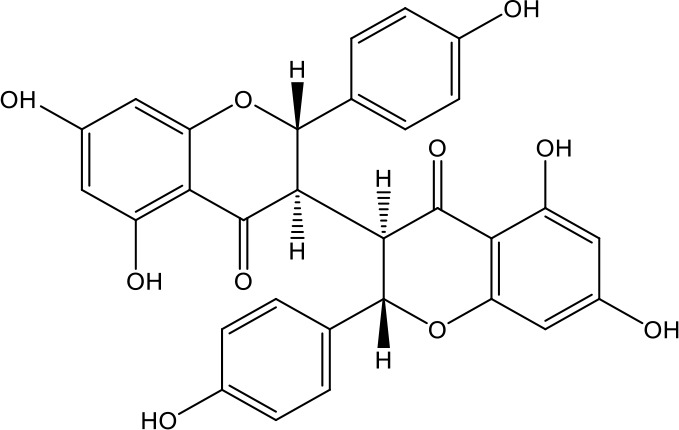	COMP2	isochamaejasmin	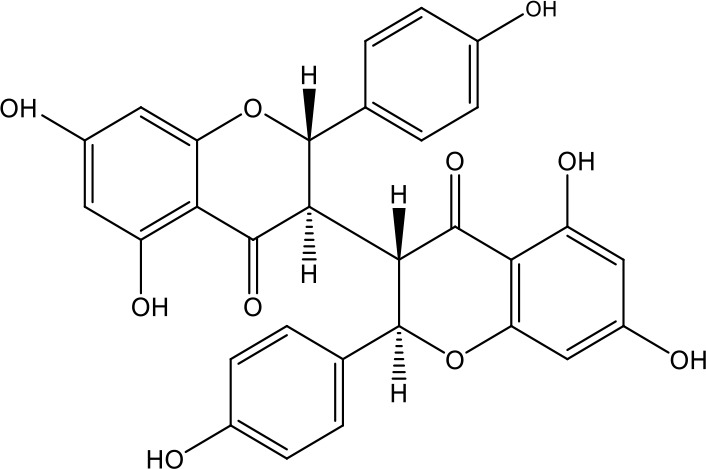
	COMP3	chamaejasmine B	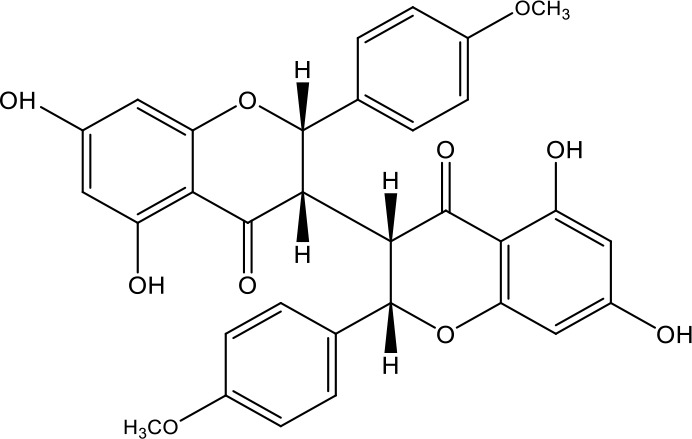	COMP4	7-methoxylneochaejasmin A	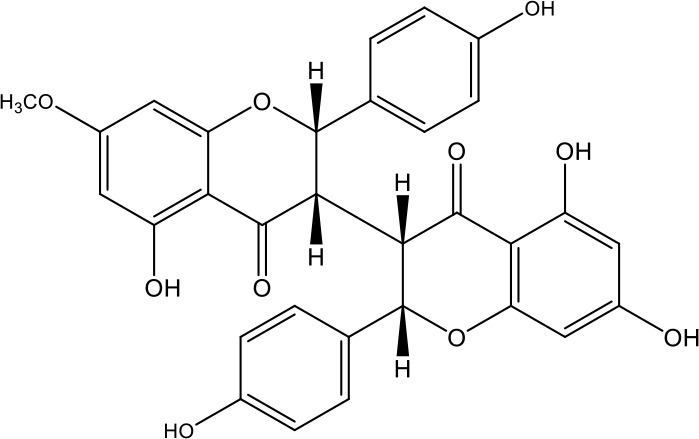
	COMP5	neochamaejasmin A	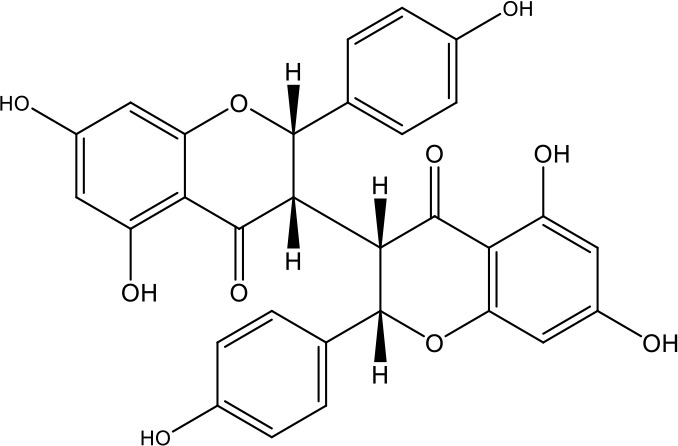	COMP6	neochamaejasmin B	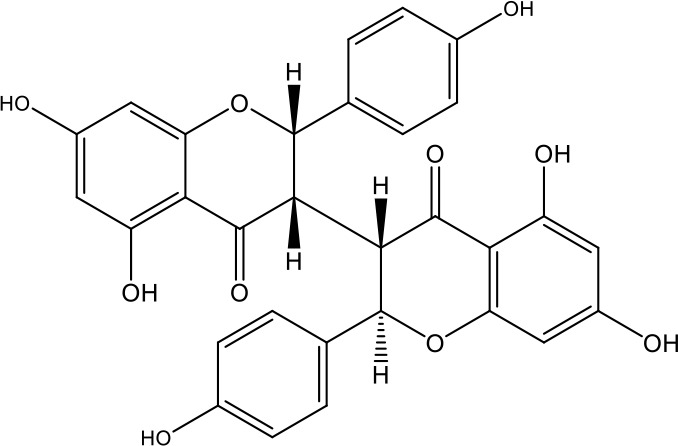
	COMP7	isoneochamaejasmin A	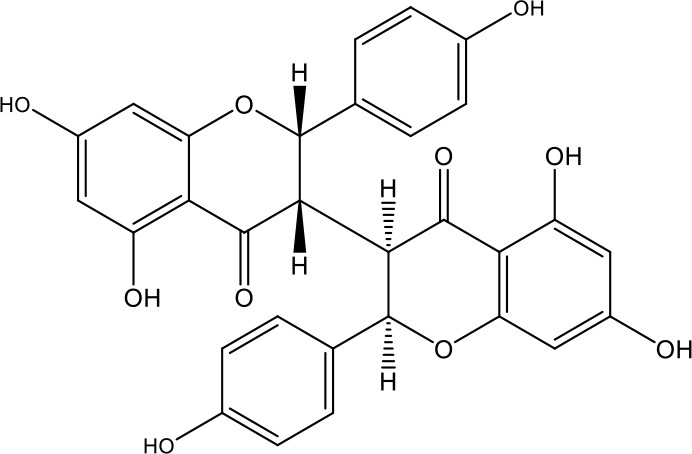	COMP8	isochamaejasmin B	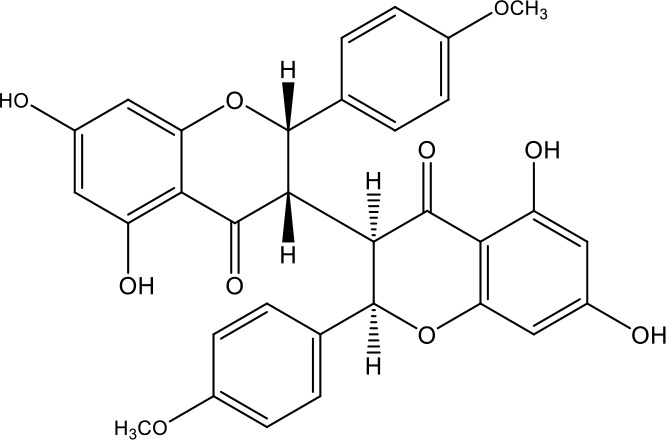
	COMP9	chamaejasmine E	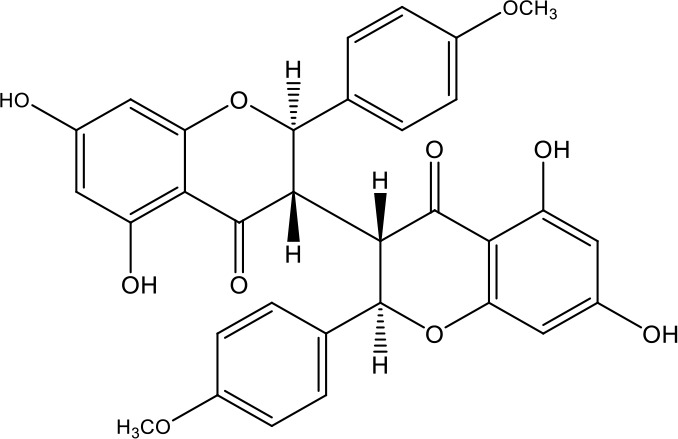	COMP10	neochamaejasmin C	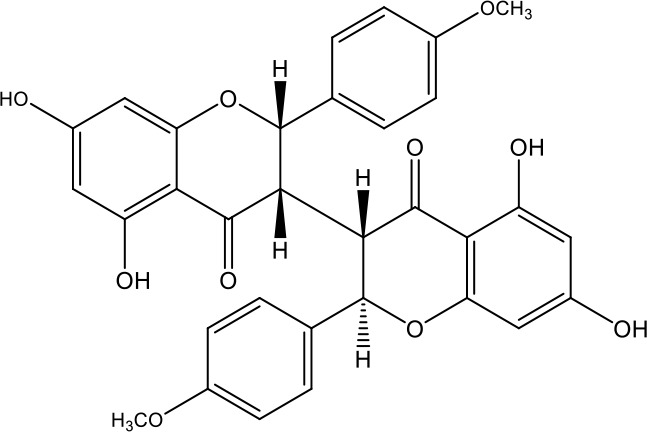
	COMP11	Ruixiang langdu A	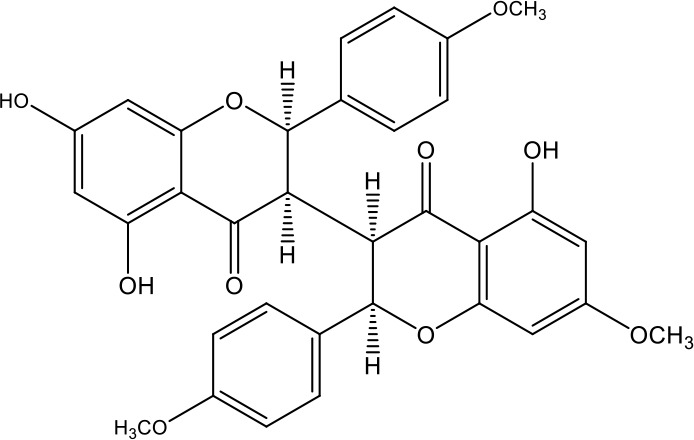	COMP12	wikstrol A	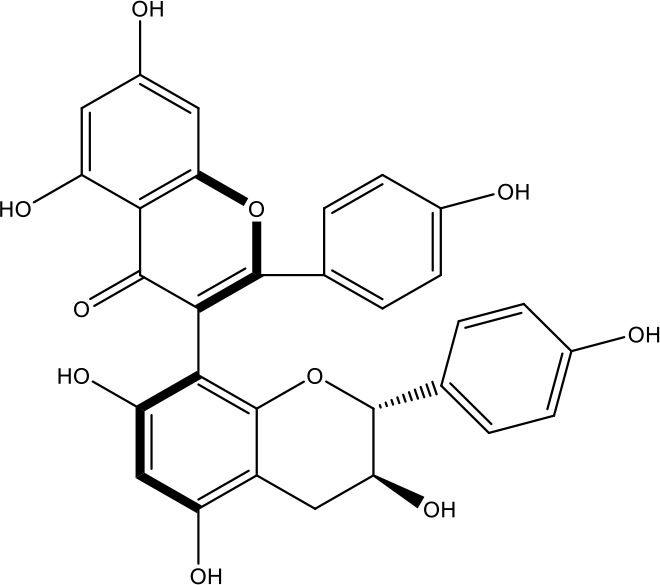
Coumarins	COMP13	daphnoretin	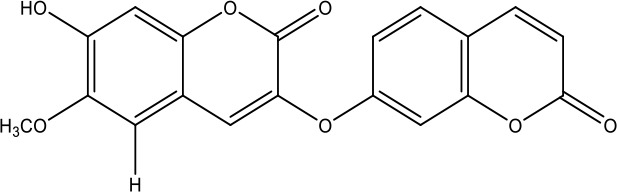	COMP14	Sphondin	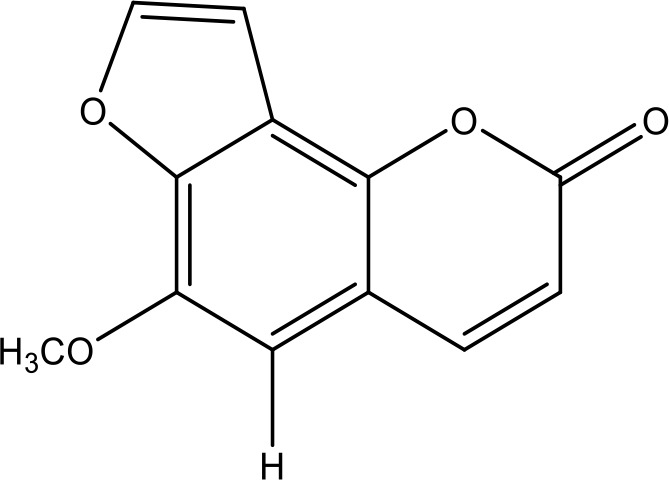
Lignans	COMP15	magnolenin C	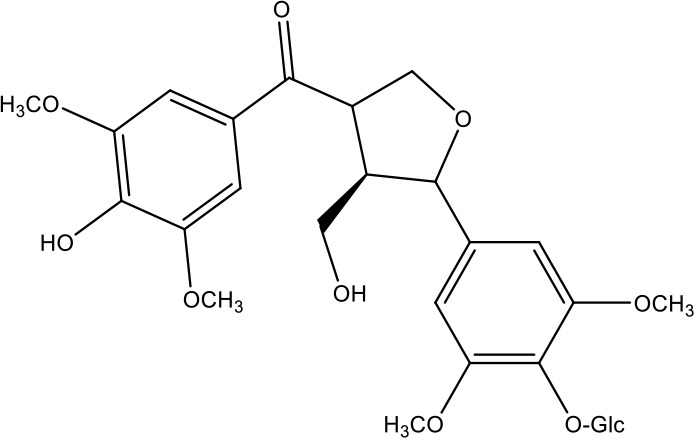	COMP16	isolariciresinol	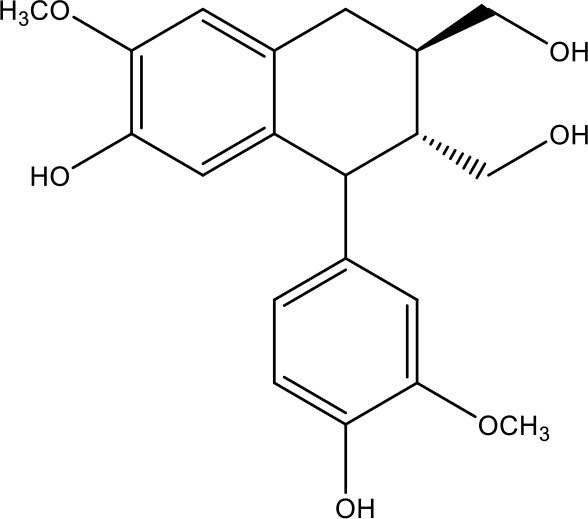
	COMP17	Syringaresinol di-O-β-D-glucopyranoside	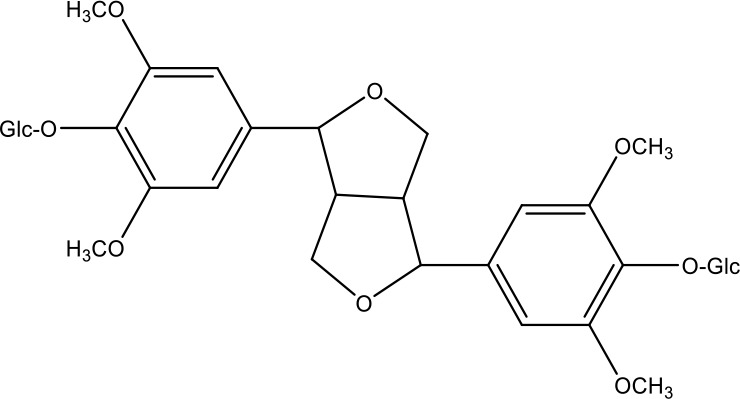	COMP18	isohinokinin	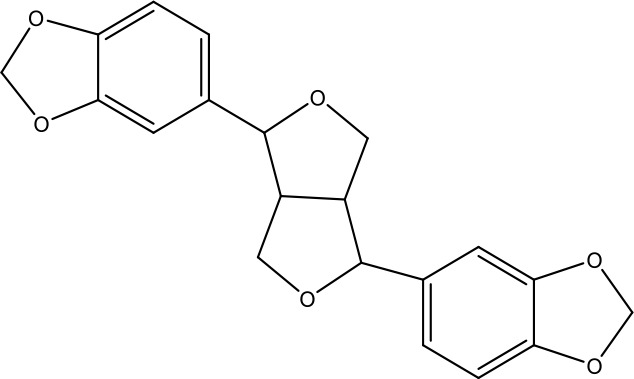
	COMP19	demethyl-trachelogenin	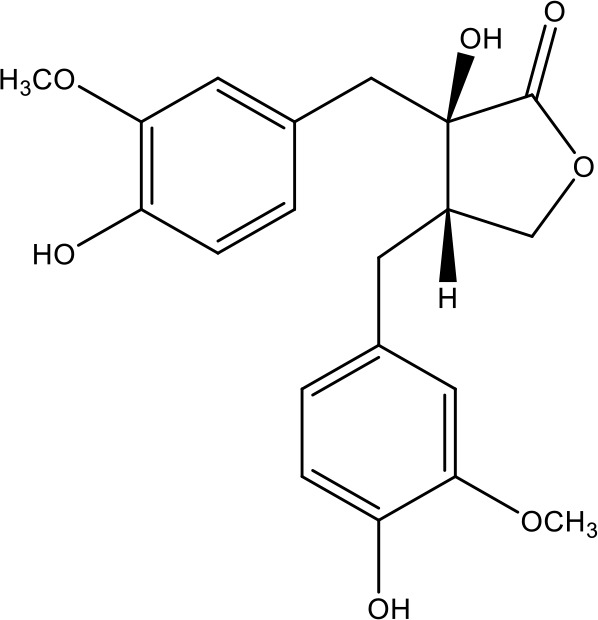	COMP20	(-) –(7R,8S,7'E)-4-hydroxy-3,5'-dimethoxy-7,4'-epoxy-8,3'-neolign-7'-ene-9,9'-diol9'-ethyl ether	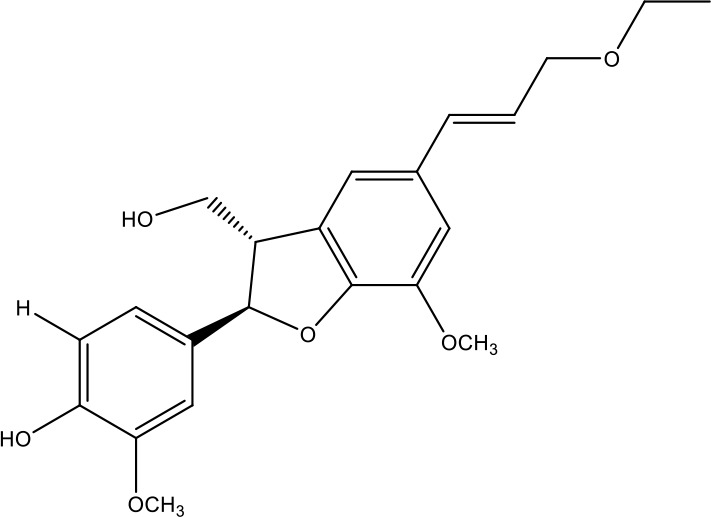
	COMP21	(-)-pinoresinol	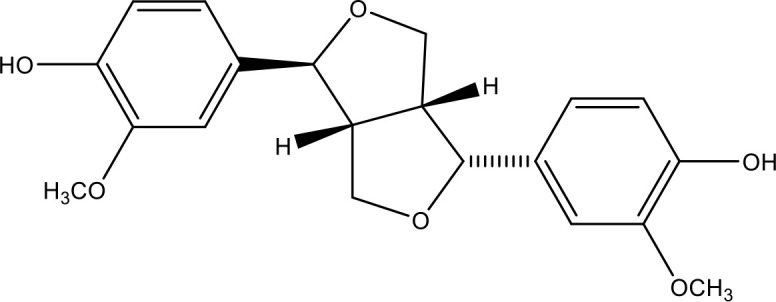			
Diterpenoids	COMP22	subtoxin A	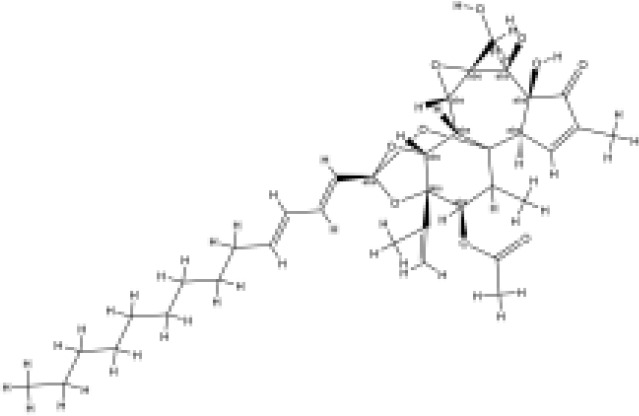	COMP23	neostellerin C	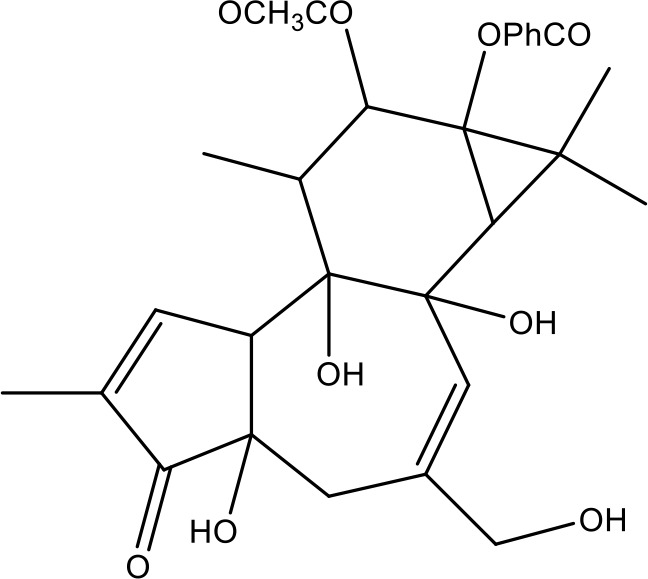
	COMP24	neostellin	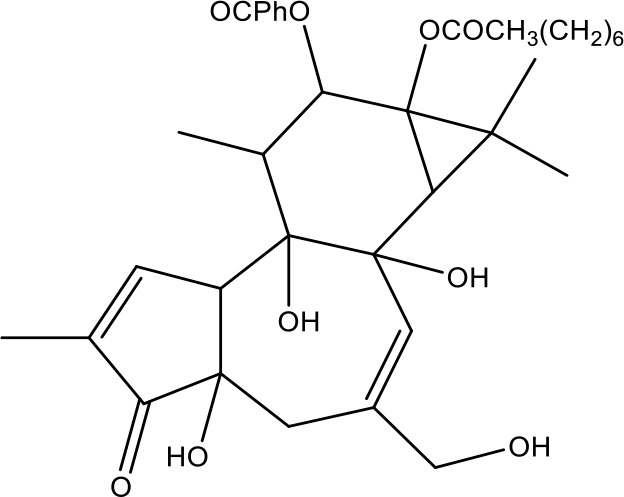	COMP25	stelleralides F	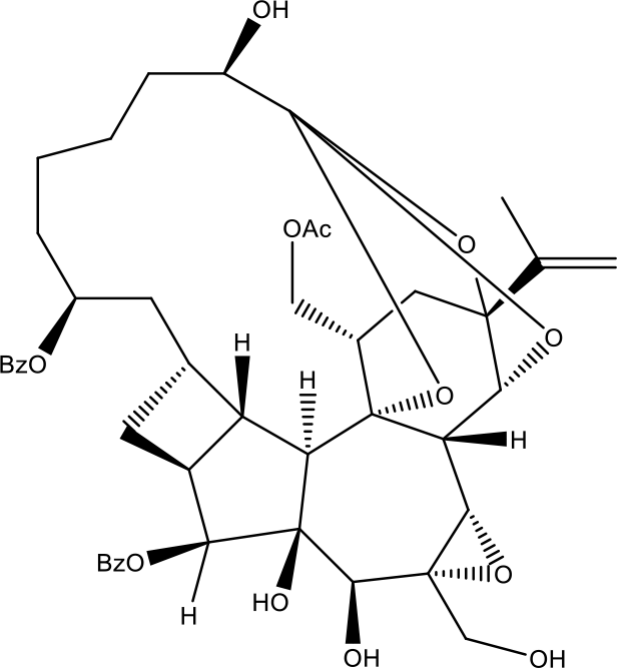
Volatile oils and other compounds	COMP26	1,5-diphenyl-1-pentanone	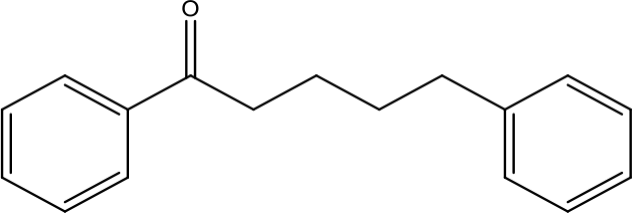			

### PPI network and CytoNCA topology analysis

**Figure 3:**
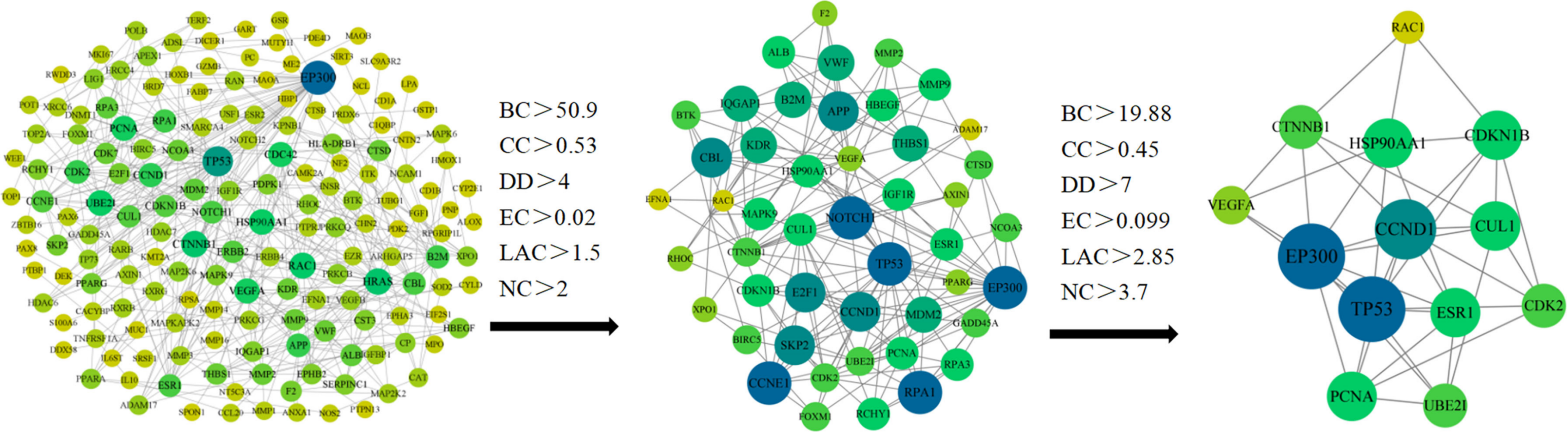
RXLD-related GBM targets’ PPI network.

We have put 216 RXLD-related GBM target genes into the String database (**Figure 3**), while the screened initial PPI network consisted of 167 nodes and 496 edges. After employing “BC > 50.9, CC > 0.53, DD > 4, EC > 0.02, the LAC > 1.5, and NC > 2” for the screening conditions (all median) criterion, the generated sub-network consisted of 51 nodes and 209 edges. The second screening was performed after employing “BC > 19.88, CC > 0.45, DD > 7, EC > 0.099, LAC > 2.85, and NC > 3.7” for the screening conditions criterion and obtained the final network that consisted of 13 nodes and 39 edges. The top 5 node degrees in the final network were TP53, EP300, VEGFA, CTNNB1, and CCND1, which we speculated as representing core targets for RXLD in treating GBM (**Supplementary 5**).

### GO and KEGG enrichment analysis

**Figure 4:**
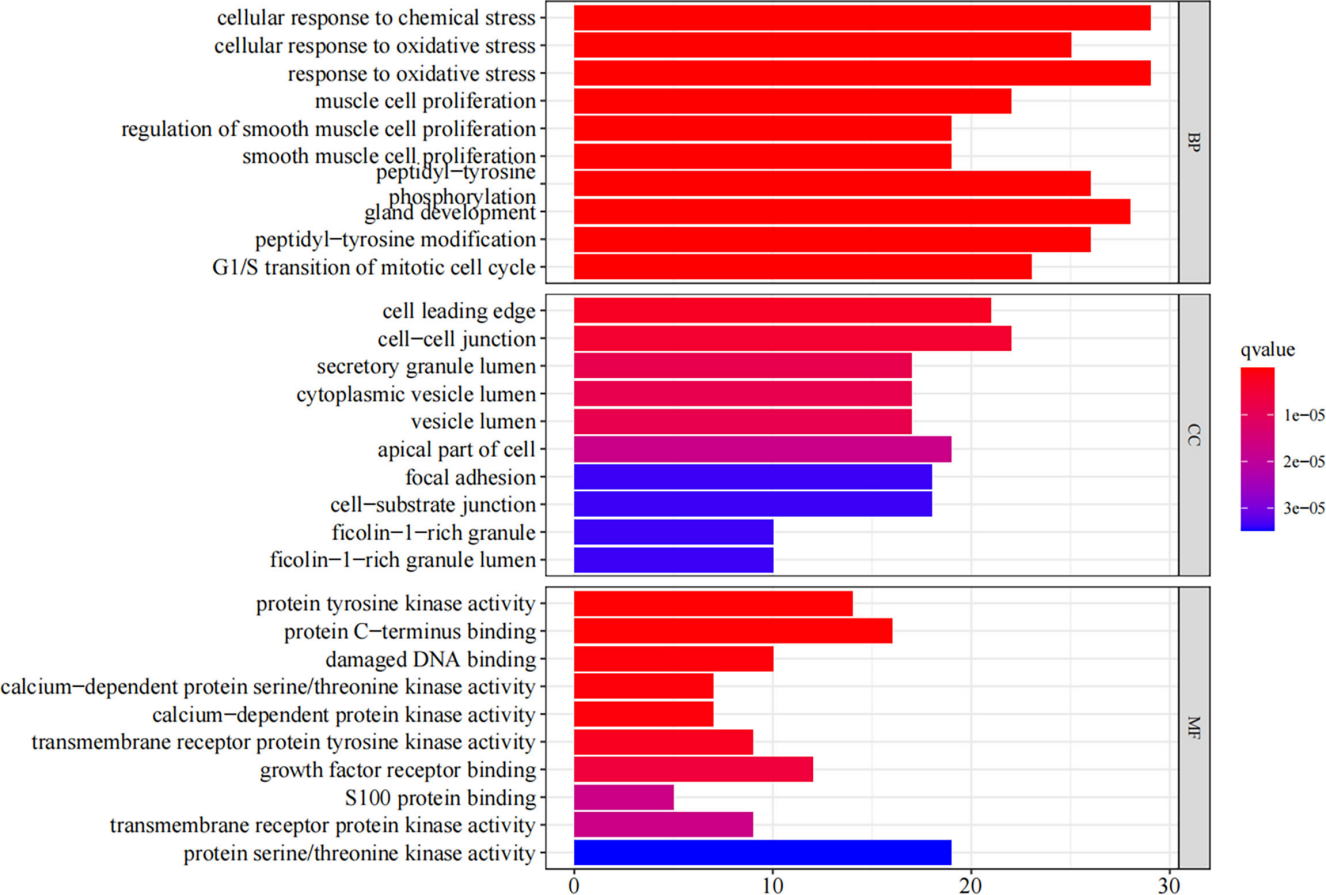
The GO enrichment analysis (BP, CC, MF) of the RXLD-related GBM targets (top 10).

We have obtained 2,035 biological processes through the GO enrichment analysis (**Supplementary 6**). **Figure 4** shows the top 10 functional categories identified, mainly involving biological processes such as oxidative stress, cell proliferation, cell cycle, cell invasion, and migration. A total of 114 signaling pathways were obtained through the undertaken KEGG enrichment analysis (**Supplementary 7**). The top 5 signaling pathways were the MAPK signaling pathway, the PI3K-AKT signaling pathway, the RAS signaling pathway, and the cell cycle, and the glioma signaling pathway (**Table 2**).

**Table 2:** KEGG enrichment analysis of the RXLD-related GBM targets (Top 10).

signaling pathway ID	Name	degree	target
hsa04010	MAPK signaling pathway	22	MAP2K2/INSR/MAPK9/IGF1R/MAPKAPK2/IRAK4/ERBB2/PRKCG/RAC1/MAP2K6/EFNA1/VEGFB/VEGFA/TP53/PRKCB/ERBB4/CDC42/FGF1/GADD45A/KDR/TNFRSF1A/HRAS
hsa04151	PI3K-Akt signaling pathway	22	MAP2K2/CCNE1/INSR/IGF1R/CCND1/HSP90AA1/ERBB2/VWF/RAC1/CDK2/EFNA1/VEGFB/THBS1/VEGFA/TP53/CDKN1B/ERBB4/FGF1/PDPK1/KDR/MDM2/HRAS
hsa04014	RAS signaling pathway	15	CALML3/MAP2K2/INSR/MAPK9/IGF1R/PRKCG/RAC1/EFNA1/VEGFB/VEGFA/PRKCB/CDC42/FGF1/KDR/HRAS
hsa04110	Cell cycle signaling pathway	14	E2F1/CCNE1/CUL1/CCND1/EP300/PCNA/CDK2/TP53/CDKN1B/SKP2/GADD45A/WEE1/MDM2/CDK7
hsa05214	GBM signaling pathway	13	CALML3/MAP2K2/E2F1/CAMK2A/CAMK4/IGF1R/CCND1/PRKCG/TP53/PRKCB/GADD45A/MDM2/HRAS
hsa04066	HIF-1 signaling pathway	13	MAP2K2/HK2/CAMK2A/INSR/IGF1R/EP300/ERBB2/PRKCG/NOS2/VEGFA/CDKN1B/PRKCB/HMOX1
hsa04210	Cell apoptosis signaling pathway	13	BIRC5/MAP2K2/GZMB/EIF2S1/MAPK9/CTSB/PTPN13/TP53/PDPK1/GADD45A/CTSD/TNFRSF1A/HRAS
hsa04218	Cell aging pathway	13	CALML3/MAP2K2/FOXM1/E2F1/CCNE1/CCND1/MAPKAPK2/CDK2/MAP2K6/TP53/GADD45A/MDM2/HRAS
hsa04310	Wnt/β-Catenin signaling pathway	12	CAMK2A/MAPK9/CUL1/CCND1/EP300/CACYBP/PRKCG/RAC1/AXIN1/TP53/PRKCB/CTNNB1
hsa04115	p53 signaling pathway	10	TP73/RCHY1/CCNE1/CCND1/CDK2/THBS1/TP53/SERPINB5/GADD45A/MDM2

**Figure 5:**
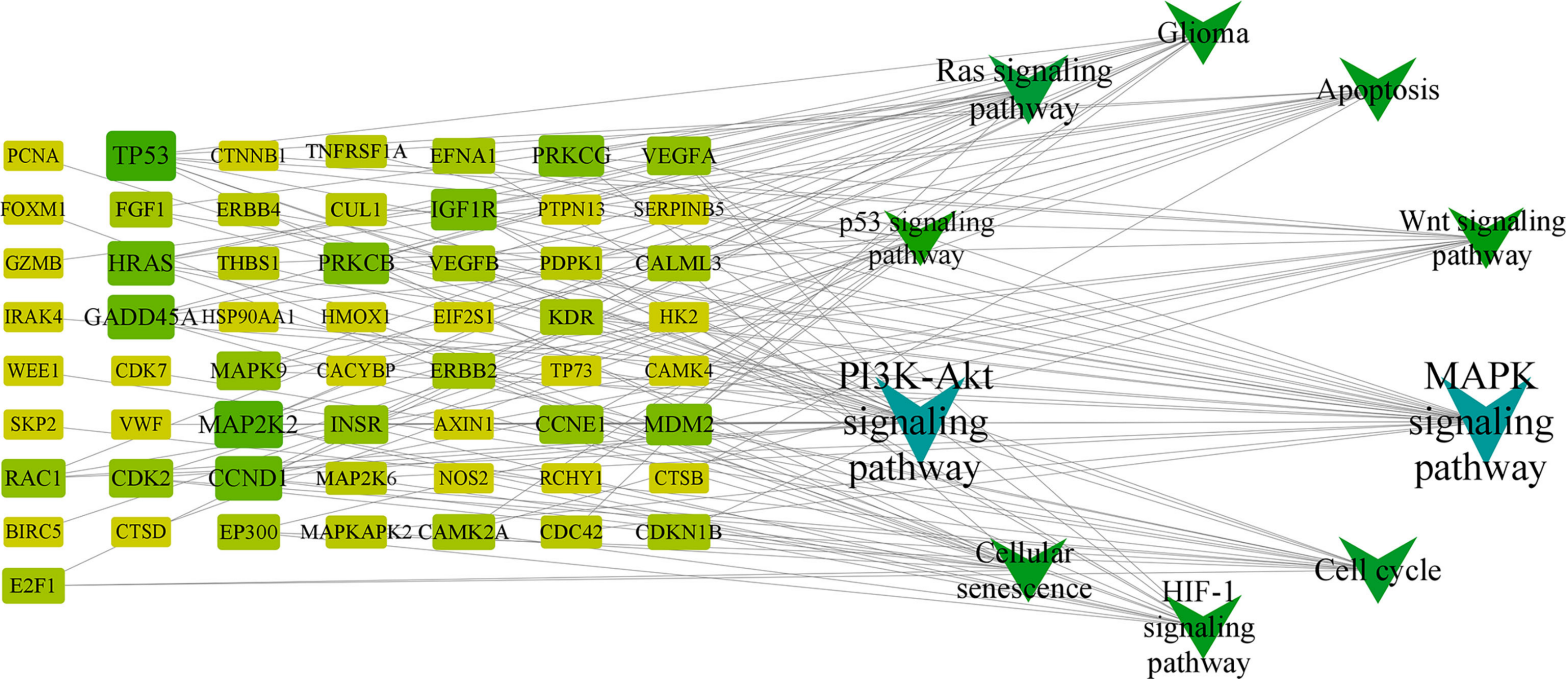
KEGG network chart of RXLD-related GBM targets.

The target-signaling pathway network was composed of 67 nodes and 147 edges, and larger nodes represented more important genes and pathways in the network. Among them, HRAS, PRKCB, MAPK9, CCND1, and TP53 were core targets in this network (**Figure 5**) (**Supplementary 8**, **9**).

### Molecular docking

**Figure 6:**
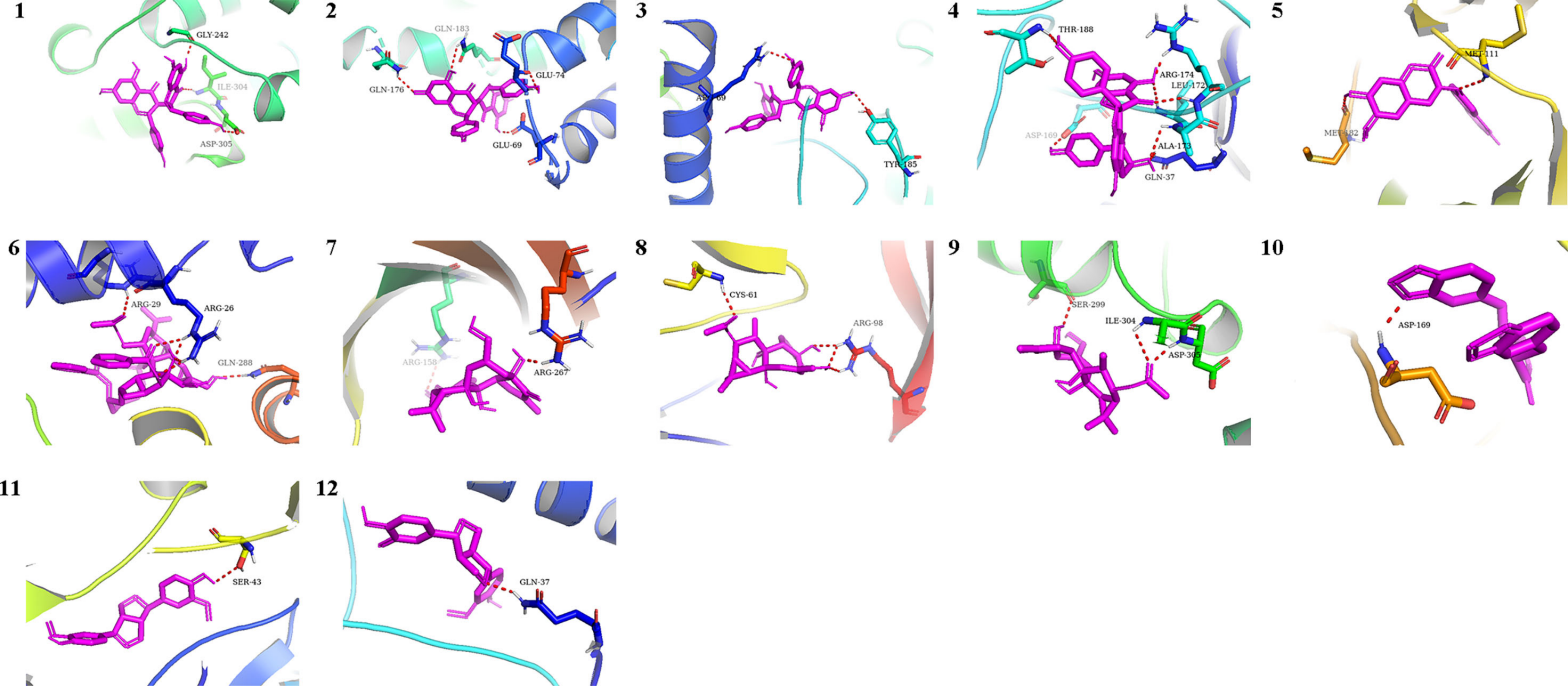
The docking analysis of core RXLD pharmacological compounds and related GBM targets. Numbers 1−12 corresponded to the docking results presented in **Table 3**.

We chose the active pharmacological compounds that demonstrated the higher degree of nodes in the RXLD-related GBM targets network as ligands and chose the core proteins that were screened in the PPI network as receptors for docking. Finally, we got 12 groups of ligand-receptor docking results (**Table 3**; **Figure 6**). The docking binding energy values of all groups were < 0 kcal/mol, indicating a spontaneously ability between ligands and receptors. Among them, 11 groups of docking binding energy values were < -7.0 kcal/mol, suggesting a strong combining ability between ligands and receptors.

**Table 3:** The docking results of core RXLD pharmacological compounds and related GBM targets.

NO.	active pharmacological compounds	Compounds number	pharmacophore model	target	Binding energy	Figure
1	isochamaejasmin	COMP2	3e7o_B_cavity_3	MAPK9	−9.3	**Figure 6–1**
2	neochamaejasmin A	COMP5	2w96_A_cavity_1	CCND1	−8.4	**Figure 6–2**
3	neochamaejasmin A	COMP5	3e7o_B_cavity_3	MAPK9	−8.0	**Figure 6–3**
4	wikstrol A	COMP12	3e7o_B_cavity_3	MAPK9	−8.3	**Figure 6–4**
5	daphnoretin	COMP13	3e7o_B_cavity_3	MAPK9	−9.0	**Figure 6–5**
6	stelleralides F	COMP25	2w96_A_cavity_1	CCND1	−8.3	**Figure 6–6**
7	neostellerin C	COMP23	2j21_A_cavity_1	TP53	−6.8	**Figure 6–7**
8	neostellerin C	COMP23	1bj1_H_cavity_1	VEGFA	−7.7	**Figure 6–8**
9	neostellerin C	COMP23	3e7o_B_cavity_3	MAPK9	−7.8	**Figure 6–9**
10	isohinokinin	COMP18	3e7o_B_cavity_3	MAPK9	−7.9	**Figure 6–10**
11	(-)-pinoresinol	COMP21	1bj1_H_cavity_3	VEGFA	−8.0	**Figure 6–11**
12	(-)-pinoresinol	COMP21	3e7o_B_cavity_3	MAPK9	−7.8	**Figure 6–12**

### RXLD extracts inhibited GBM cell proliferation and induced apoptosis

**Figure 7:**
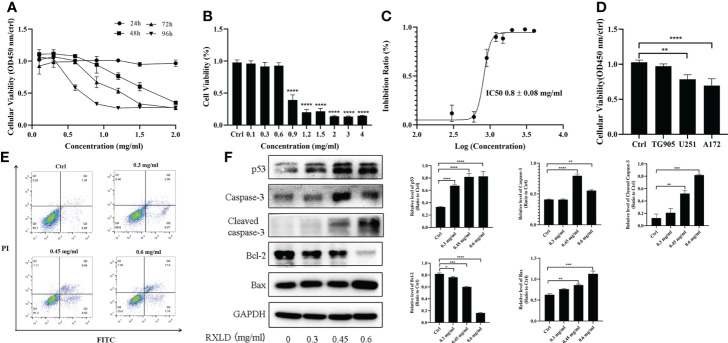
RXLD extracts inhibited GBM cell proliferation and induced apoptosis. **(A)** Cell viability of the A172 glioma cells after treatment with difference concentrations of RXLD extracts for 24, 48, 72, and 96 h; **(B, C)** Cell viability of the A172 glioma cells after treatment with difference concentrations of RXLD extracts for 48 h; **(D)** Cell viability of the A172, TG905, and U251 cell lines after incubation with RXLD extracts (0.8 mg/ml); **(E)** RXLD extracts with different concentrations can induce apoptosis in the A172 cell line; **(F)** RXLD extracts with different concentrations can affect the levels of apoptosis-related genes (p53, caspase-3, cleaved caspase-3, Bcl-2, and Bax) in A172 cells. These analyses were repeated three times. * P<0.05, ** P<0.01, *** P< 0.001, **** P<0.0001.

Through the cell proliferation assay, we found that the RXLD-induced cell proliferation inhibition was time- and dose-dependent (**Figure 7A**) in the A172 cell line, while the IC50 was 0.8 ± 0.08 mg/ml (**Figures 7B, C**). When we treated the GBM cell lines (A172, TG905, and U251) with 0.8 mg/ml of RXLD extracts, we found that the cell viability of the U251 and A172 cell lines were significantly decreased (P < 0.01) (**Figure 7D**); among them, the decline of the cell viability of the A172 cell line was the most evident, and this is why the A172 cells were used in the subsequent experiments. The annexin V-FITC/PI apoptosis experiments showed that the apoptosis rate was time- and dose-dependent (**Figure 7E**), while the levels of the apoptosis-related genes p53, cleaved-caspase3, and Bax were found to be gradually increased, and the Bcl-2 levels were gradually decreased (*P*< 0.01) (**Figure 7F**). As a result, the RXLD extracts were shown to inhibit the proliferation of GBM cells and to induce apoptosis.

### RXLD extracts blocked the GBM cell cycle in the G2/M and S phases, and can inhibit the Wnt/β-catenin pathway

**Figure 8:**
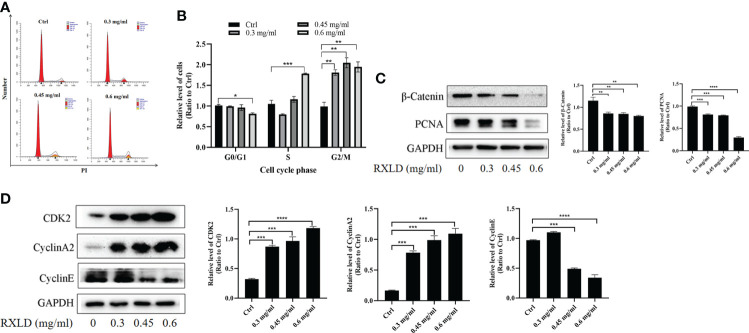
RXLD extracts blocked the GBM cell cycle in the G2/M and the S phases, and can inhibit the Wnt/β-catenin pathway. **(A, B)** Cell cycle analysis of the A172 cells treated with RXLD extracts (0.3, 0.45, and 0.6 mg/ml) for 48 h, with DNA fragmentation being observed; **(C, D)** Western blotting results of cell cycle-related genes (β-catenin, PCNA, cyclin E, CDK2, and cyclin A2) expressed in A172 cells that were treated with RXLD extracts (0.3, 0.45, and 0.6 mg/ml) for 48 h. * P<0.05, ** P<0.01, *** P< 0.001, **** P<0.0001.

When we treated the A172 cell line with RXLD extracts, we found that as the drug concentration increased, the number of GBM cells in the G0/G1 phase decreased in the cell cycle assay, while the number of GBM cells in the S and G2/M phases increased. The difference was statistically significant (*P*< 0.001; **Figures 8A, B**). Western blotting experiments suggested that the protein levels of β-catenin, proliferating cell nuclear antigen (PCNA), and cyclin E were gradually declining. The cyclin-dependent kinase 2 (CDK2) and cyclin A2 protein levels gradually increased (**Figures 8C, D**). The above results indicate that the RXLD extracts block the cell cycle in the G2/M and the S phases, thereby affecting GBM cells’ proliferation through the Wnt/β-catenin pathway.

### RXLD extracts suppressed the migration of GBM cells and regulated the expression of proteins related to migration

**Figure 9:**
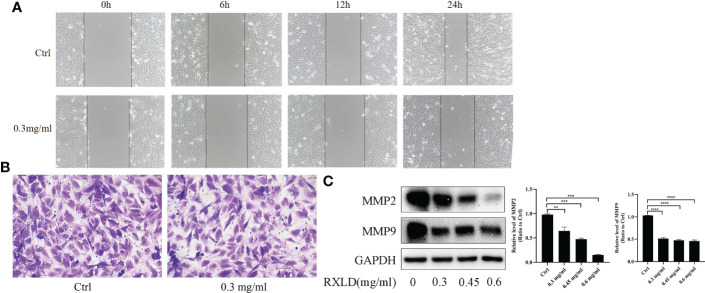
RXLD extracts affected the migration of GBM cells. **(A)** The wound healing assay was performed on the A172 cell line at 0, 6, 12, and 24 h after the cells were treated with RXLD extracts; **(B)** The cell migration results (transwell assay) of the A172 cell line that was treated with RXLD extracts for 48 h; **(C)** Western blotting results of the cell migration-related genes (MMP2, MMP9) expressed in A172 cells that were treated with RXLD extracts (0.3, 0.45, and 0.6 mg/ml) for 48 h. ** P<0.01, *** P<0.001, **** P<0.0001.

The results of the cell wound healing and the cell migration experiments revealed that RXLD extracts (0.3 mg/ml) could inhibit the migration of A172 cells (**Figures 9A, B**). Western blotting experiments indicated that as the drug concentration increased, the protein expression levels of matrix metalloproteinase 2 (MMP2) and MMP9 gradually declined (*P*< 0.01; **Figure 9C**). These results indicate that the RXLD extracts can inhibit GBM cell migration.

### RXLD extracts regulated the oxidative stress and the ferroptosis process of GBM cells and inhibited p62/Nrf2

**Figure 10:**
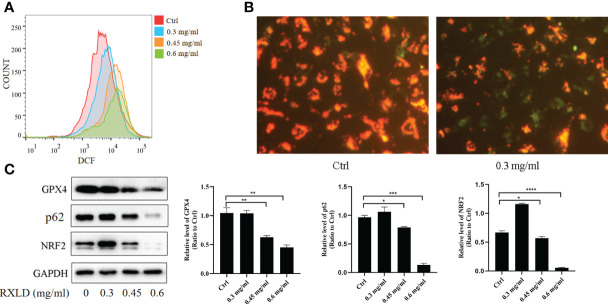
RXLD extracts regulated the oxidative stress and the ferroptosis process of GBM cells and inhibited p62/Nrf2 pathway. **(A)** The ROS assay was performed on A172 cells that were treated with RXLD extracts for 48 h; **(B)** The mitochondrial membrane potential assay was performed on A172 cells that were treated with RXLD extracts for 48 h; **(C)** Western blotting results of the p62 signaling pathway-related genes (GPX4, P62, and NRF2) in A172 cells treated with RXLD extracts (0.3, 0.45, and 0.6 mg/ml) for 48 h. * P<0.05, ** P<0.01, *** P< 0.001, **** P<0.0001.

The ROS experiments revealed that the DCF peak was significantly moving toward the right after the A172 cells were treated with RXLD extracts (compared to the control group), suggesting that the intracellular reactive oxygen levels increased (**Figure 10A**). JC-1 is a fluorescent probe used to detect mitochondrial membrane potential (ΔΨm). The decrease of ΔΨm is a hallmark event in the early stages of apoptosis and related to ferroptosis. Compared with the control group, the green fluorescence levels increased significantly after the A172 cells were treated with 0.3 mg/ml of RXLD extracts (**Figure 10B**), suggesting that the mitochondrial membrane potential decreased and that the cells were going through an early apoptosis stage. Western blotting experiments showed that the protein expression levels of glutathione peroxidase 4 (GPX4), p62, and nuclear factor-erythroid factor 2-related factor 2 (NRF2) genes gradually decreased with the increase of the drug concentrations used (*P* < 0.001; **Figure 10C**). These findings show that the RXLD extracts can regulate the oxidative stress of A172 cells through the p62/Nrf2 pathway and can induce ferroptosis in GBM cells.

## Discussion

In this study, we first constructed the pharmacophore models of the RXLD chemical components. We then screened the chemical compositions with the better effects according to the rule “Normfit≧ 0.8.” The screening results suggested that flavonoids, lignans, and diterpenoids among the chemical components of RXLD were the main active RXLD components in the treatment of GBM; these three classes of components were worthy of further studying in order to explore their pharmacological mechanisms. Through the RXLD-related GBM targets’ network analysis, we could identify neochamaejasmin A (COMP5), wikstrol A (COMP12), subtoxin A (COMP22), isochamaejasmenin (COMP2), and chamaejasmine (COMP1). They all demonstrated higher degrees of interaction that indicate that they might be the main pharmacological components of RXLD with the potential of being useful in the treatment of GBM. Yang *et al. (* 25) found that chamaejasmine can induce autophagy and the production of active oxygen in osteosarcoma MG-63 cells by activating the AMPK pathway and by inhibiting the mTOR pathway. In breast cancer, chamaejasmine can induce expression of p21 and p27, and block the cell in the G2/M phase as well as promote apoptosis by phosphorylating NF-κB in MDA-MB-231 cells (26). The 3D structure of the isochamaejasmenin was highly similar to that of the Bcl-2 ligand (-)-gossypol, which can induce apoptosis in human leukemia HL-60 and K562 cells *via* the Bcl-2 cell apoptosis pathway (27). On the other hand, neochamaejasmin A has been shown to promote ROS-related mitochondrial apoptosis *via* the phosphorylation of the Erk1/2/JNK signaling pathway in hepatoblastoma HepG2 cells (28). Finally, wikstrol A can induce apoptosis in esophageal carcinoma Eca-109 cells and block their cell cycle in the S phase by up-regulation of the peroxisome proliferator-activated receptor-gamma (PPARγ) expression (29).

To further clarify the specific mechanisms through which the RXLD extracts could treat GBM, we employed the GO enrichment analysis. We found that RXLD is primarily involved in oxidative stress, cell proliferation, cell cycle, cell invasion, and migration processes. Therefore, we used a variety of cell function experiments to verify the above biological functions by using the RXLD extracts to treat the GBM cells. Our experimental results revealed that RXLD extracts could induce apoptosis, block cell cycle in the G2/M and the S phases, regulate the cell cycle, affect cell migration (through inhibition of the Wnt/β-catenin pathway), and regulate oxidative stress and ferroptosis (via the p62/Nrf2 pathway) in GBM cells.

Subsequently, we used the RXLD-related GBM targets’ network, the PPI network, and the KEGG network for further screening, and we eventually identified some core targets of RXLD for the treatment of GBM (such as HRAS, PRKCB, MAPK9, CCND1, TP53, etc.). We then screened 12 active RXLD compounds through molecular docking by using the above targets. Among the above targets, the HRAS gene was responsible for encoding H-RAS protein (30), which is highly related to the malignant phenotype of the GBM cell line (31).MAPK was HRAS’s downstream pathway that participates in the apoptosis, proliferation, autophagy, invasion, and metastasis of the tumor cells (32).In this cascading reaction, the induced apoptosis is a result of the phosphorylation of key regulatory factors (such as TP53, YAP1) and the PKC-mediated cell procedural death (33, 34), while the blocked TP53 ubiquitination increases its stability in non-stress cells (35); moreover, MAPK9 can also promote the degradation of β-catenin/CTNNB1 and can inhibit the classic Wnt signaling pathway (36), thereby further adjusting the downstream genes CCND1, MMP2, MMP9 in order to affect the proliferation and migration of the cells (37, 38). MAPK9 plays an important role in the regulatory biological clock by phosphorylating the ARNTL/BMAL1 heterodimers (39). Transcription factor Nrf2 plays an important role in cellular oxidative stress (40), and its transcriptional activation has been related to anti-ferroptosis (41). Nrf2 overexpression can cause chemical-sensitive tumor cells to resist ferroptosis inducers by inhibiting KEAP1 overexpression (42). Moreover, the expression of the selective autophagy cargo adaptor SQSTM/P62 can prevent Nrf2 from degradation and enhance its nuclear accumulation through the deactivation of the KEAP1 protein (43). In addition, SQSTM1/P62 can act as a cargo receptor responsible for the autophagic degradation of the clock protein ARNTL. The latter is known to inhibit ferroptosis through the repression of the transcription of Egln2 and the activation of the pro-survival transcription factor HIF1A to promote lipid peroxidation (44). In summary, the RAS/MAPK pathway may act as an upstream pathway during the GBM treatment process with RXLD, and it might further affect the regulation of the Wnt/β-catenin, the oxidative stress, and the ferroptosis pathways.

## Conclusions

We have herein identified neochamaejasmin A (COMP5), wikstrol A (COMP12), subtoxin A (COMP22), isochamaejasmenin (COMP2), and chamaejasmine (COMP1) as the main hits of the RXLD-related GBM targets’ network analysis. We also confirmed that RXLD extracts can induce apoptosis and block the cell cycle at the G2/M and the S phases. They can also affect cell proliferation and cell migration by inhibiting the Wnt/β-catenin pathway and regulating the oxidative stress and ferroptosis pathways (through the p62/Nrf2 pathway). Finally, we have speculated that the RAS/MAPK may be an upstream pathway utilized by RXLD in treating GBM, as indicated by the RXLD-related GBM targets’ network, the PPI network, and the KEGG network screening results.

This study provides the foundations for further research toward the clinical application of traditional Chinese medicine RXLD in GBM treatment. We will focus on the pharmacological mechanisms of individual ingredients identified in RXLD, particularly those presenting good docking scores against the MAPK9 target.

## Data Availability Statement

The datasets presented in this study can be found in online repositories. The names of the repository/repositories and accession number(s) can be found in the article/**Supplementary Material**.

## Author contributions

LW and FC designed the research. KW completed the network pharmacology work and part of the biological experiments. ZYW, XX, and ZQW assisted in part of the *in vitro* experiments. LZ and KW drafted the manuscript. All authors wrote, read, assisted in the revision, and approved the manuscript.

## Funding

This work was supported by the China Postdoctoral Science Foundation (number 2018M632687), Natural Science Foundation of Shandong Province (number ZR2021QH231), Key Research Project program of Shandong Province (number 2019GSF108088), Traditional Chinese Medicine Technology Development Project of Shandong Province (number 2019-0852), Medical Technology Development Project of Shandong Province (number 202102040561), Linyi Science and Technology Development Project (number 202020001).

## Conflict of interest

The authors declare that the research was conducted in the absence of any commercial or financial relationships that could be construed as a potential conflict of interest.

## Publisher’s note

All claims expressed in this article are solely those of the authors and do not necessarily represent those of their affiliated organizations, or those of the publisher, the editors and the reviewers. Any product that may be evaluated in this article, or claim that may be made by its manufacturer, is not guaranteed or endorsed by the publisher.
